# Daily living skills scale: Development and preliminary validation of a new, open-source assessment of daily living skills

**DOI:** 10.3389/fpsyt.2022.1108471

**Published:** 2023-01-23

**Authors:** Mirko Uljarević, Emily K. Spackman, Ru Ying Cai, Katherine J. Paszek, Antonio Y. Hardan, Thomas W. Frazier

**Affiliations:** ^1^Department of Psychiatry and Behavioral Sciences, School of Medicine, Stanford University, Stanford, CA, United States; ^2^School of Psychological Sciences, University of Melbourne, Melbourne, VIC, Australia; ^3^Aspect Research Centre for Autism Practice, French's Forest, Sydney, NSW, Australia; ^4^Department of Psychology, John Carroll University, University Heights, OH, United States

**Keywords:** assessment, daily living skills, adaptive functioning, neurodevelopmental, neuropsychiatric, autism

## Abstract

Autistic individuals and individuals with a range of other neurodevelopmental conditions (NDD) often present with lower levels of daily living skills (DLS) when compared to their neurotypical peers. Importantly, lower levels of DLS have been linked to a range of negative outcomes, including lower rates of post-secondary education, lower employment rates, and higher daily support needs across autism and NDD. However, there are currently no open-source informant-reported instruments for capturing key aspects of DLS. This study describes the development, refinement, and initial psychometric evaluation of a new, relatively brief (53-item). Daily Living Skills Scale (DLSS) in a sample of 1,361 children aged 2–17 years, Confirmatory Factor Analysis demonstrated an excellent fit of unidimensional model to the data (CFI = 0.953, TLI = 0.951, RMSEA = 0.073 [95% CI: 0.071–0.074]). The single-factor CFA model showed evidence of measurement invariance of factor loadings, thresholds, and residual variance (strict invariance) across sex, age, race, and ethnicity. Model reliability and internal consistency were excellent (ω = 0.98; α = 0.97). Conditional reliability estimates indicated very good reliability (= 0.80) for the total DLS scale from very low (θ = −4.2) to high (θ = +2.4) scores. Conceptually derived self-care, homecare, and community participation subscales also showed strong reliability and internal consistency. With further replication, the EFS has excellent potential for wide adoption across research and clinical contexts.

## 1. Introduction

In addition to core diagnostic characteristics, autistic individuals and individuals with a range of other neurodevelopmental conditions (NDD), including, but not limited to, attention-deficit hyperactivity disorder (ADHD), intellectual disabilities (ID), and neurogenetic conditions, often present with lower levels of daily living skills (DLS) when compared to their neurotypical peers (1–4). Importantly, lower levels of DLS have been linked to lower rates of post-secondary education, lower employment rates, increased likelihood of living with parents, and higher daily support needs in individuals with NDD, in particular in autistic individuals with and without co-occurring ID (5–7). Conversely, higher DLS are predictive of more positive adult outcomes, including better subjective wellbeing (8) and higher education rates, employment, and independent living (9). Consequently, DLS have been recognized as a crucial target for clinical intervention and support by both research and clinical community as well as by autistic individuals and stakeholders (10–12).

DLS are typically acquired across development and are defined as the ability to perform the everyday tasks and activities necessary for independent living. They fall into three main domains: personal (e.g., dressing, showering, and taking medication), domestic (e.g., cooking, cleaning, and laundry), and community DLS [e.g., managing time, money, and employment; (13)]. Autistic individuals and individuals with other NDD demonstrate significant heterogeneity in these skills that cannot be accounted for by cognitive functioning alone (1, 14, 15). In fact, although higher cognitive functioning is broadly associated with higher DLS (16), autistic individuals with average or above average IQs often show considerably lower DLS than would be expected given their cognitive functioning, whereas DLS has been identified as a relative strength of many individuals with co-occurring ID (17–21). Further, although some core autism characteristics, such as communication barriers or rigid routines, may play a role in the reduced acquisition of these skills, research has shown that the autism characteristics alone are poor predictors of DLS (1, 3, 21, 22). Therefore, DLS represent a distinct and crucial target for more individualized research and support that is stakeholder-informed and reflects a shift toward a more dimensional and trans-diagnostic understanding of support needs.

Although current measures of DLS, with the Vineland Adaptive Behavior Scale [VABS-3; (13)] and the Adaptive Behavior Assessment System [ABAS-3; (23)] being the most widely adopted instruments, have provided important insights into presentations of DLS across neurodevelopmental conditions, they are not without limitations. Firstly, although relatively comprehensive, both instruments are lengthy and can be burdensome for participants, limiting their use in clinical contexts and research studies with longer assessment batteries. Further, high cost prohibits their use among research and clinical communities with less funding or could decrease the collection of adaptive functioning in situations where money must be allocated to more expensive measurement types (e.g., multi-omic data collections). Therefore, despite stakeholder calls to better contextualize autism research in terms of specific challenges requiring support, the expense and length of current instruments could preclude the inclusion of DLS assessments from being more widely adopted. Additionally, the current measures of DLS have demonstrated psychometric limitations. For example, despite the complexity and noted heterogeneity of DLS (1, 9, 13, 16, 21), most commonly used measures either provide only a total DLS score based solely on theoretical or practical concerns, limiting the clinical utility of these measures in terms of identifying specific targets for intervention and monitoring the progress of specific skills, or have not been comprehensively and stringently factorized, raising concerns about their construct validity. Additionally, measurement invariance and factor structures of current measures are largely unexplored and remain poorly understood, despite their wide use. This is a significant issue given that measurement invariance is crucial for ensuring that a measure is applicable across a wide demographic spectrum. Further, several studies evaluating VABS psychometric properties have reported low internal consistency for subscale scores as well as low interrater and test-retest reliability (13, 24–26). Finally, despite increasing recognition of the utility of regression-based norms to improve the precision and individualization of clinical assessment (27), current measures do not provide regression-based norms across autistic, neurotypical, and other developmental disorder populations. Thus, there is an urgent need for new, freely-available, comprehensive DLS instruments developed and validated based on state-of-the-art measurement development and psychometric principles.

The current paper outlines the development and psychometric evaluation of the Daily Living Skills Scale (DLSS)—a relatively brief (53-items) yet comprehensive, open-source informant-reported instrument for capturing key aspects of DLS that was specifically developed to be appropriate for use across full developmental (age and cognitive functioning) and demographic range and to be applicable across a range of NDD. DLSS was designed to specifically address the limitations of current DLS measures by providing a robust factor structure and measurement invariance across key parameters, including age, sex, and diagnostic status. The development process included a literature review of current DLS measures and item evaluation by stakeholders. Preliminary validation was conducted with a large, representative US sample spanning autistic children, children with other NDD, and neurotypical controls and included evaluation of the factor structure, measurement invariance, classical test theory and item response theory-derived reliability, and convergent and discriminant validity.

## 2. Materials and methods

### 2.1. Participants

Parent informants were recruited using the Prolific online data collection service (https://prolific.co/). Data was collected from 05/03/2022 to 07/20/2022. The final sample included 1,361 informants who completed the survey on their children. Based on informant-reported clinical diagnoses, 116 children had autism, 356 had neurodevelopmental conditions, and 889 were neurotypical (no neurodevelopmental or neuropsychiatric diagnosis). Inclusion criteria for both exploratory and confirmatory samples included: residence in the US, having a dependent child aged 2–17, and informant proficiency in English. Detailed characteristics across exploratory and confirmatory samples are presented in Table 1.

**Table 1:** Demographic and clinical characteristics across autism spectrum disorder (ASD), developmental disability (DD), and neurotypical (NT) controls.

	**NT**	**DD**	**ASD**	***X^2^/F* (*p*)**
	***n*** **(%)**	***n*** **(%)**	***n*** **(%)**	
*N*	889	356	116	
Informant (*n*, %)				32.16 (<0.001)
Biological mother	508 (57.1%)	250 (70.2%)	79 (68.1%)	
Biological father	380 (42.7%)	106 (29.8%)	36 (31.0%)	
Other/not reported	1 (0.2%)	0 (0%)	1 (0.9%)	
Informant age (M, SD)	40.97 (9.29)	41.97 (9.07)	39.09 (9.06)	13.56 (<0.001)
Highest parental education (*n*, %)				26.8 (0.003)
Less than HS	6 (0.6%)	2 (0.6%)	1 (1.0%)	
High school or GED	90 (8.9%)	38 (10.8%)	11 (10.6%)	
Some college	178 (17.6%)	94 (26.8%)	32 (30.8%)	
College graduate	427 (42.2%)	132 (37.6%)	40 (38.5%)	
Graduate degree or higher	295 (29.2%)	80 (22.8%)	18 (17.3%)	
Unknown	16 (1.6%)	5 (1.4%)	2 (1.9%)	
US region				10.9 (0.205)
Northeast	188 (18.6%)	51 (14.5%)	16 (15.4%)	
Midwest	215 (21.3%)	69 (19.7%)	23 (22.1%)	
South	402 (39.8%)	168 (47.9%)	50 (48.1%)	
West	203 (20.1%)	62 (17.7%)	15 (14.4%)	
Other/chose not to respond	4 (0.4%)	1 (0.3%)	0 (0.0%)	
Household income (*n*, %)				43.1 (<0.001)
<$25,000	74 (8.3%)	37 (10.4%)	17 (14.7%)	
$25,000–$34,999	87 (9.8%)	41 (11.5%)	15 (12.9%)	
$35,000–$49,999	96 (10.8%)	49 (13.8%)	19 (16.4%)	
$50,000–$74,999	191 (21.5%)	72 (20.2%)	25 (21.6%)	
$75,000–$99,999	152 (17.1%)	61 (17.1%)	14 (12.1%)	
$100,000–$149,999	182 (20.5%)	64 (18.0%)	11 (9.5%)	
$150,000–$199,999	50 (5.6%)	14 (3.9%)	9 (7.8%)	
$200,000 and above	50 (5.6%)	13 (3.7%)	6 (5.2%)	
Unknown	0 (0.0%)	5 (1.4%)	0 (0.0%)	
Child age (M, SD)	8.8 (4.6)	11.6 (4.3)	10.05 (4.5)	49.7 (<0.001)
Child biological sex (*n*, % male)	433 (48.7%)	183 (51.4%)	88 (75.9%)	34.11 (<0.001)
**Race**				
White/Caucasian (*n*, %)	705 (81.0%)	292 (82.0%)	94 (79.3%)	1.2 (0.537)
Black/African American (*n*, %)	97 (10.9%)	27 (7.6%)	17 (14.7%)	5.5 (0.062)
Middle eastern (*n*, %)	4 (0.4%)	1 (0.3%)	1 (0.9%)	0.7 (0.712)
East Asian (*n*, %)	34 (3.8%)	7 (2.0%)	3 (2.6%)	2.9 (0.226)
South Asian (*n*, %)	16 (1.2%)	2 (0.1%)	0 (0.0%)	4.7 (0.096)
Pacific Islander (*n*, %)	6 (0.7%)	0 (0.0%)	0 (0.0%)	3.2 (0.202)
Native American (*n*, %)	9 (1.0%)	9 (2.5%)	2 (1.7%)	4.1 (0.129)
Unknown race (*n*, %)	2 (0.2%)	1 (0.3%)	0 (0.0%)	0.3 (0.849)
Chose not to respond (*n*, %)	9 (1.0%)	2 (0.6%)	0 (0.0%)	0.3 (0.868)
Hispanic or Latino (*n*, %)	101 (11.4%)	43 (12.1%)	26 (22.4%)	12.4 (0.015)
**Non-ASD diagnoses (** ***n*** **, %)**				
ID/GDD	–	10 (2.8%)	6 (5.8%)	2.1 (0.150)
Speech/language disorder	–	75 (21.4%)	16 (15.5%)	1.7 (0.193)
ADHD	–	146 (41.6%)	29 (27.9%)	6.1 (0.014)
ODD/CD	–	25 (7.1%)	5 (4.9%)	0.7 (0.415)
Anxiety disorder	–	111 (31.6%)	19 (18.4%)	6.8 (0.009)
Specific learning disorder	–	33 (9.4%)	3 (2.9%)	4.6 (0.032)
Motor/coordination disorder	–	16 (4.6%)	2 (1.9%)	1.4 (0.231)
Depressive disorder	–	50 (14.2%)	8 (1.8%)	3.0 (0.083)
Bipolar disorder/mania	–	7 (2.0%)	1 (1.0%)	0.5 (0.488)
Obsessive compulsive disorder	–	11 (3.1%)	5 (4.9%)	0.7 (0.405)
Tic disorder	–	6 (1.7%)	1 (1.0%)	0.3 (0.593)
Feeding/eating disorder	–	16 (4.6%)	0 (0.0%)	4.9 (0.029)

NT, neurotypical controls; DD, non-ASD developmental disability; ASD, autism spectrum disorder; ID/GDD, intellectual disability/global developmental delay; ADHD, attention-deficit/hyperactivity disorder; ODD/CD, oppositional defiant disorder/conduct disorder. Non-ASD diagnoses do not sum to 100% because children could be diagnosed with more than one condition. Cognitive level information was only completed for n = 886.

### 2.2. Measures

Daily Living Skills Scale (DLSS). DLSS was developed and refined through an iterative series of steps embodied in the Patient-Reported Outcomes Measurement Information System (PROMIS) framework. Firstly, the DLS conceptual model was generated based on a systematic review of the existing DLS instruments and existing conceptual models of both broader adaptive functioning and more specific DLS frameworks. The following key content areas were identified: Personal Hygiene and Grooming, Dressing and Undressing, Meal Preparation and Feeding, Toileting, Housekeeping, Health and Medication, Leisure Time, Safe Environment, Transportation and Mobility. Following the generation of the conceptual map, a review of the literature was conducted to identify existing scales relevant to each content area. Reviewed scales included: the Vineland Adaptive Behavior Scales, third edition [VABS-3; (13)], the Adaptive Behavior Assessment System [ABAS-3; (23)], the Scales of Independent Behavior—Revised [SIB-R; (28)], and Waisman Activities of Daily Living [W-ADL; (29)]. Instruments were then reviewed by the first and senior author, and at least three items were written to ensure that each content area is adequately assessed with particular emphasis on capturing the full ability range, from very low to high (capturing very high levels was deemed not crucial for most clinical and research contexts). A preliminary item bank was evaluated by 10 neurodevelopmental disability clinician-scientist experts and 10 neurodevelopmental disability caregiver/patient informants. Both experts and caregivers/patient informants evaluated whether each item (i) effectively evaluated the given DLS content area (experts and informants), (ii) was relevant to patients (experts) or child (informants), (iii) was relevant to the full developmental (age and IQ) and functional range of patients (experts), and (iv) was easy/difficult to understand and rate (experts and informants). Following the feedback, minor clarifications and changes in terms of the wording were made to several items. Given that neither experts nor caregivers/patient informants indicated that any of the items should be removed and that no specific behaviors, skills, or content areas were missing, no further changes were made to the scale. The final scale consisted of 53 items that were rated on a 4-point Likert scale with the choices being: “Not able to complete (total assistance needed),” “Requires significant prompting or assistance,” “Requires minimal prompting or assistance,” “Completely independent (does not require any assistance or prompting)”. This scale was chosen given that being able to differentiate between significant prompting/assistance and only minimal prompting/assistance should add discrimination to each item and is consistent with behavioral intervention approaches that attempt to decrease the level of prompting needed when building functional skills. No time frame was included in the instructions because daily living skills are rated according to whether the skill has been acquired at the time of rating.

Demographic and health information. Informants provided age, sex, race, ethnicity, time spent living with the informant, household income, and highest level of parental education, and reported all prior clinical diagnoses for each participant.

Vineland Adaptive Behavior Scales, third edition [VABS-3; (13)]. VABS-3 is an informant-report scale designed to comprehensively capture different aspects of adaptive functioning. Each item is rated on a 3-point Likert scale (0 = Never, 1 = Sometimes, 2 = Usually), with higher rating indicating better performance/ability/skill level. For the present study, the total Daily Living Skills domain (143 items) score was used. VABS-3 Daily Living Skills domain encompasses Personal, Domestic, and Community subdomains. For the present study, the total domain score was used.

DSM-5 attention-deficit/hyperactivity disorder (ADHD) Checklist. DSM-5 ADHD Checklist is an 18-item informant report scale designed to capture DSM-5 ADHD symptoms of inattention, hyperactivity, and impulsivity. Each item is rated on a 4-point Likert scale (0 = Not at all; 1 = Just a Little; 2 = Often; and 3 = Very Often) with a higher rating indicating more severe symptoms.

Developmental Coordination Disorder Questionnaire [DCDQ; (30)]. DCDQ is a 15-item informant-report measure designed to capture fine and gross motor skills as well as general coordination in children and adolescents. Each item is rated on a 5-point Likert scale, with higher scores reflecting better performance. Total score was used in this study.

Spence Children's and Preschool Anxiety Scales [SCAS and SPAS; (31, 32)]. SCAS and SPAS are informant-report measures of anxiety designed to capture overall anxiety levels and specific anxiety subdomains. Each item is rated on a 4-point Likert scale (0 = Neve, 1 = Sometimes, 2 = Often, and 3 = Always) where higher rating indicates higher symptom severity. The SPAS was administered to ages 2–6 and the SCAS was administered to ages 7–17. The total scores were used for the current study.

Executive Functioning Skills scale [EFS; (33)]. EFS is a 52-item scale designed to comprehensively characterize non-affective (e.g., sequencing/working memory, response inhibition and set-shifting) and affective (e.g., emotion regulation, and risk-taking) facets of executive functioning. Total EFS score is strongly correlated with the 24-item Behavior Rating Inventory of Executive Functioning (*r* = 0.85) and shows excellent model reliability and internal consistency (ω = 0.98; α = 0.97) and excellent conditional reliability estimates (≥0.90) from extremely low (θ ~ −4.2) to very high (θ ~ +2.6) scores.

### 2.3. Statistical analyses

Descriptive statistics for demographic and clinical factors were computed to characterize the sample.

#### 2.3.1. Factor structure

Confirmatory Factor Analysis was first used to explore the fit of the unidimensional model. Model fit was evaluated using the comparative fit index (CFI), Tucker–Lewis index (TLI), root mean square error of approximation (RMSEA), and the 95% confidence interval of RMSEA were used to examine model fit (34, 35). If the unidimensional model showed poor fit, we planned to estimate exploratory structural equation models [ESEM; (36)] using geomin rotation and weighted least squares mean and variance adjusted estimation and specifying two to 8 specific factors with an additional general bifactor that included estimation of loadings from all items.

#### 2.3.2. Measurement invariance

The optimal model derived from the factor analyses described above was used as the basis for the evaluation of measurement invariance (37) across age groups (ages 2–4, 5–11, and 12–17 years), sex (male, female), race (Caucasian, other), and ethnicity (Hispanic, non-Hispanic). To examine measurement invariance (equivalence), a series of multi-group confirmatory factor analyses were computed using the theta parameterization and WLSMV estimation for categorical indicators, following recommended conventions (38) and our prior work (39). Model comparisons for measurement invariance analyses were based on empirical work indicating that a drop in CFI or TLI > 0.01 or an increase in RMSEA > 0.01 implies measurement non-equivalence (40, 41).

#### 2.3.3. Reliability

Using the optimal factor model, items with substantive loadings were assigned to scales and classical test theory (CTT) reliability (internal consistency and correct item-total correlations) (42) and item response theory (IRT) analyses were conducted (43) in the full sample (*n* = 2004). Analyses used maximum likelihood estimation with robust standard errors and a logit link with the single factor mean and variance fixed to 0 and 1, respectively. Reliability estimates falling in the ranges 0.70–0.79, 0.80–0.89, and >0.90 were considered fair, good, and excellent (44). Average corrected item-total correlations ≥0.30 were considered adequate or better (42). Differential item and test functioning were evaluated by examining differences in item characteristic curves and test information curves across age groups, sex, race, and ethnicity.

#### 2.3.4. Convergent and discriminant validity

Convergent and discriminant validity were computed using bivariate correlations (Pearson or Spearman's non-parametric, where applicable).

## 3. Results

### 3.1. Factor structure and measurement invariance

**Figure 1:**
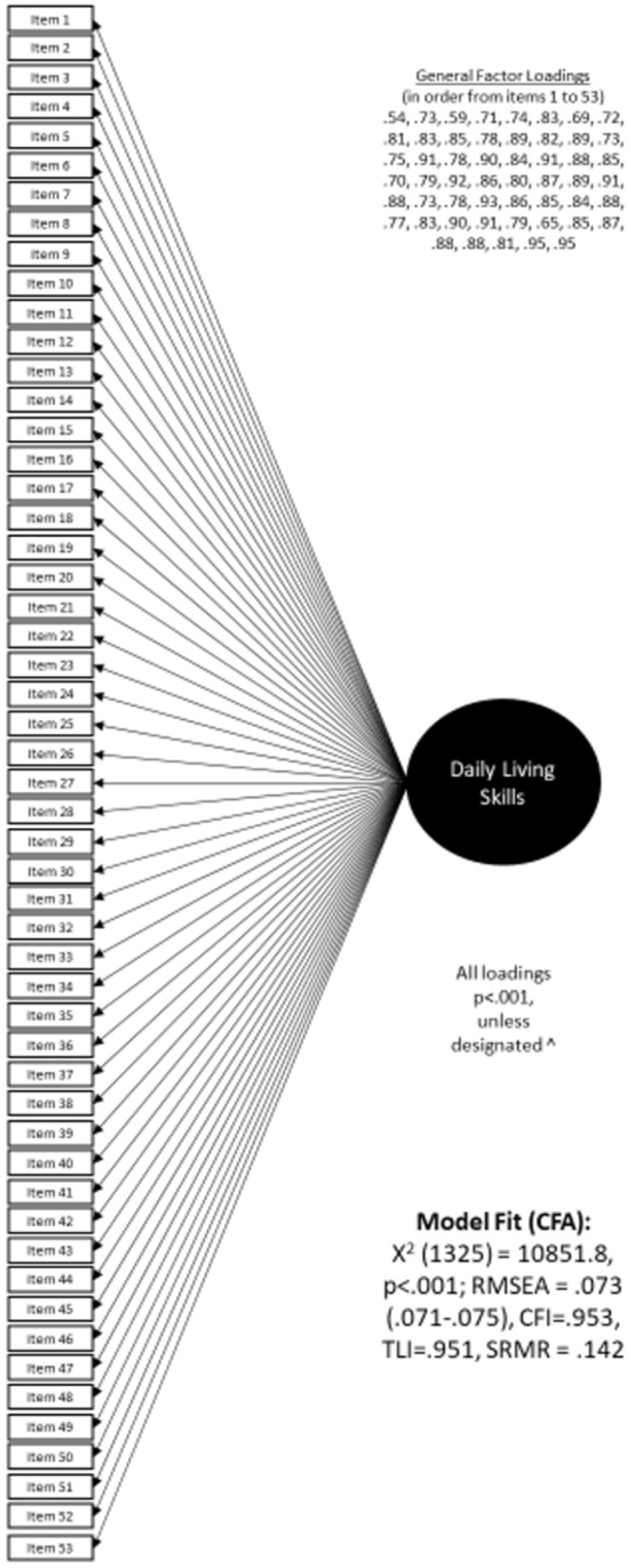
Single factor CFA model in the total validation sample.

The CFA single-factor model based on the final 53 items showed good fit to the data (CFI = 0.953, TLI = 0.951, RMSEA = 0.073 [95% CI: 0.071–0.074]). All items had strong loading on the single factor (Figure 1). The single-factor CFA model was used to evaluate measurement invariance, model reliability, and variance accounted for by the specific and general DLS factors. Given that other daily living skills (Vineland-3) or practical domain (ABAS-3) measures include content subareas and that there is value in helping clinicians understand what types of skills might be strengths or weaknesses, we also computed three subscales: self-care (28 items), home-care (10 items), and community participation (15 items). Scale reliability information is also provided for these conceptual subscales.

The single-factor CFA model presented above showed evidence of measurement invariance of factor loadings, thresholds, and residual variance (strict invariance) across sex, age, race, and ethnicity (Table 2).

**Table 2:** Measurement invariance analyses for the final DLS factor model across sex, age, race, and ethnicity.

	**Fit**						**Difference testing**					
**Model**	**Par**	*X* ^2^	**DF**	**RMSEA**	**CFI**	**TLI**	*X* ^2^	**DF**	***p***	Δ**RMSEA**	Δ**CFI**	Δ**TLI**
**Sex (M, F)**												
Configural	424	11,869.5	2,650	0.072	0.954	0.952	–	–	–	–	–	–
Metric	372	11,882.5	2,702	0.071	0.954	0.953	58.5	52	0.2479	−0.001	0.000	0.001
Scalar	267	11,950.8	2,807	0.069	0.954	0.955	183.5	105	<0.0001	−0.002	0.000	0.002
**Age (2–4, 5–11, 12–17)**												
Configural	636	16,447.0	3,975	0.083	0.845	0.838	–	–	–	–	–	–
Metric	532	16,533.2	4,079	0.082	0.845	0.843	512.1	104	<0.0001	−0.001	0.000	0.005
Scalar	322	16,698.3	4,289	0.080	0.845	0.851	915.4	210	<0.0001	−0.002	0.000	0.008
**Race (Caucasian, other races)**												
Configural	424	11,410.4	2,650	0.070	0.954	0.953	–	–	–	–	–	–
Metric	372	11,387.0	2,702	0.069	0.955	0.954	66.9	52	0.0797	−0.001	0.001	0.001
Scalar	267	11,389.3	2,807	0.067	0.955	0.956	125.9	105	0.0806	−0.002	0.000	0.002
**Ethnicity (hispanic, non-hispanic)**												
Configural	424	11,617.6	2,650	0.071	0.955	0.953	–	–	–	–	–	–
Metric	372	11,538.1	2,702	0.069	0.956	0.955	42.4	52	0.8272	−0.002	0.001	0.002
Scalar	267	11,518.5	2,807	0.068	0.957	0.957	110.3	105	0.3419	−0.001	0.001	0.002

### 3.2. Model and scale reliability

Model reliability was high for the general DLS factor (ω = 0.98) and comparable to the Vineland-3 Daily Living Skills domain reliability in this sample (ω = 0.99). Using item scores, internal consistency reliability was excellent for the total scale (α = 0.97) and comparable to the Vineland-3 Daily Living Skills domain (α = 0.99) in this sample. Internal consistency reliability of the conceptual scores was also excellent (self-care α = 0.96; home-care α = 0.94; community participation α = 0.94).

### 3.3. Conditional reliability derived from item response theory analyses

**Figure 2:**
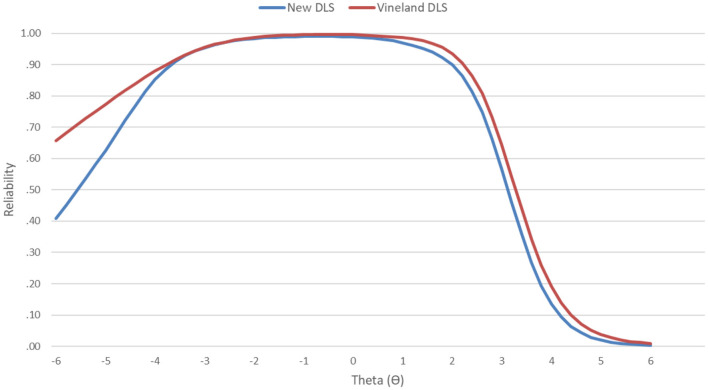
Item response theory-derived conditional reliability across the latent trait for the total DLS scale and the Vineland Daily Living Skills domain.

Conditional reliability estimates indicated very good reliability (≥0.80) for the total DLS scale from very low (θ = −4.2) to high (θ = +2.4) scores. Importantly, a comparison of conditional reliability from the 53-item DLS scale and the 143-item Vineland-3 Daily Living Skills domain in this sample indicated highly comparable curves (Figure 2). The average difference in conditional reliability from theta = −4 to +4 was trivial (Δ*r*_*xx*_ = 0.02). This indicates that the DLS is showing good precision of measurement across key score ranges for monitoring intervention progress (equivalent to standard scores of 37–136) and that the DLS and Vineland Daily Living Skills domain are showing equivalent measurement precision in this sample.

### 3.4. Convergent and discriminant validity

The expected pattern of convergent and discriminant validity was observed. More specifically, DLS total scores showed strong correlations with VABS-3 Total Daily Living Skills domain raw scores (*r* = 0.94) and the DLS and Vineland-3 Daily Living Skills domain subscale scores showed the expected strong correlations (Self-Care with Personal: *r* = 0.88; Home-Care with Domestic: *r* = 0.88; Community Participation with Community: *r* = 0.91). The DLS total score showed a moderate and expected negative correlation with ASD diagnosis (*r* = −0.22) and IQ (*r* = −0.22). As anticipated, correlations with other measures that do not assess daily functioning but that evaluate clinical domains which could reduce adaptive function were also in the moderate range (DSM-5 ADHD scale *r* = −0.35; Spence Children's Anxiety Scale *r* = −0.40, Spence Children's Anxiety Scale *r* = −0.03). Not surprisingly, given the importance of executive functioning for daily living skills and the close relationship between motor functioning and daily living skills, the correlations between DLS total scores and measures of these constructs were higher (DLS with EFS *r* = 0.59 and DLS with DCDQ *r* = −0.75), however, correlations with these measures were significantly lower than correlations with the Vineland-3 Daily Living Skills scores as indexed by the Fisher *r*-to-*z* transformation (for DLS with EFS vs. Vineland-3 *z* = 27.63, *p* < 0.001; for DLS with DCDQ vs. Vineland 3 *z* = 19.94, *p* < 0.001).

## 4. Discussion

The DLSS is an open-source, informant-report measure that was developed to comprehensively assess key facets of daily living skills (DLS) across the full ability and cognitive range in children and adolescents with autism, other neurodevelopmental conditions and across normative development. Findings from this preliminary validation study demonstrate that the DLSS has excellent psychometric properties, which combined with the fact that it free and relatively brief (53 items), suggest that it is potentially a good choice for capturing DLS across different contexts, both in terms of research and potentially clinical practice. Crucially, despite having slightly more than a third of the items compared to the Vineland Adaptive Behavior Scales, third edition (DLSS = 53 vs. Vineland DLS domain = 143 items), DLSS shows equivalent reliability, internal consistency, and measurement precision.

The DLSS was developed to capture a range of different daily living skills that could be broadly divided into self-care, homecare, and community participation subdomains and encompass a diverse range of skills and behaviors, including personal hygiene and grooming, dressing and undressing, meal preparation and feeding, toileting, housekeeping, health and medication, leisure time, safe environment, and transportation and mobility. In keeping with other DLS measures such as Vineland-3, latent variable modeling indicated that the unidimensional model showed an excellent fit to the data, indicating a unidimensional factor structure. Further, a single-factor model showed evidence of measurement invariance of factor loadings, thresholds, and residual variance (strict invariance) across sex, age, race, and ethnicity. Model reliability and internal consistency were high, and conditional reliability estimates indicated very good reliability for the total DLS scale from very low to high scores. Finally, there was good preliminary evidence for convergent and discriminant validity. Although self-care (28 items), homecare (10 items), and community participation (15 items) subscales were conceptually-derived as analogs to Vineland-3 subscales, they nevertheless showed strong reliability and internal consistency. Thus, given in-depth coverage of these DLS subareas, the DLSS might be useful in helping clinicians understand types of skills that might be strengths or weaknesses for a particular child. Future studies are needed to better understand the potential value of these conceptually-derived subscales.

Although several DLS measures, including ABAS, SIB-R, and Vineland-3, have been specifically designed and widely used for capturing individual differences in DLS among autistic individuals and individuals with a range of neurodevelopmental conditions, their limited psychometric evaluation, together with significant length and cost, limit their utility. More specifically, demonstrated invariance across diverse sex, age, race, ethnicity, and clinical groups is a key assumption that must be met for widespread measure adoption of any instrument. However, there is little evidence for the invariance of other DLS scales, including ABAS, SIB-R and Vineland-3. In contrast, as noted, the DLSS showed strong evidence for invariance, indicating that it can be interpreted consistently when implemented across populations with diverse developmental levels and demographics. Good conditional reliability is a key feature necessary for capturing and tracking very high and very low levels of a particular trait with good precision; however, with the exception of Vineland-3, other measures of daily living skills lack evidence for conditional reliability. Conversely, DLSS conditional reliability estimates indicated very good measurement precision across key score ranges for monitoring intervention progress. Thus, robust evidence of invariance and conditional reliability provide tentative support for DLSS as being more useful for assessing adaptive behavior in many research contexts where brevity, rater burden, equivalent measurement across demographic groups, and cost are key considerations.

Despite a large, representative validation sample, stringent analytical approaches and comprehensive convergent and discriminant validity indicators, the findings reported here need to be considered in the light of several limitations. In particular, this study was limited by the reliance on informant reports and the lack of direct clinical diagnostic, cognitive, and symptom severity assessments. Further, even though online data collection does not allow independent confirmation of diagnostic status and administration of gold standard diagnostic assessments and dedicated cognitive assessments, it is important to note that prior online studies collecting parent-reported diagnoses have shown very high rates of ASD verification from clinical reports (45, 46) and high concordance (>97%) with clinician best estimate diagnoses and with standardized instruments (47). Further, with regards to cognitive functioning, parent-reported levels of children's IQ/cognitive functioning level have been shown to strongly correspond with in-person IQ testing [e.g., (48)]. In addition, even though the Vineland-3 DLS section was used, a more comprehensive set of additional measures is needed. Despite the large sample size, our cohort included a mainly white and well-educated sample. Given the noted limitations, it will be crucial to further validate the DLSS in a more diverse sample and clinical settings through in-person clinical and cognitive assessments and utilize longitudinal and treatment designs to investigate its predictive validity and sensitivity to change. Finally, it will be important to develop regression-based norms that take into account age, sex, and developmental and cognitive level.

In summary, despite noted limitations, the present data provide preliminary evidence that the DLSS is a valid and reliable new, freely available and relatively brief instrument for the comprehensive characterization of individual differences in different facets of DLS in autism and a range of other NDD. Further, DLSS shows excellent measurement precision for capturing a wide range of abilities, which suggests the excellent potential for its use for characterizing change over time and for treatment tracking. Thus, with further replication, the DLSS has excellent potential for wide adoption across research and clinical contexts.

## Data availability statement

The raw data supporting the conclusions of this article will be made available by the authors, without undue reservation. And I did not detect any particular expressions.

## Ethics statement

The studies involving human participants were reviewed and approved by John Carroll University IRB. Written informed consent to participate in this study was provided by the participants' legal guardian/next of kin.

## Author contributions

TF, MU, and AH designed the study. TF and MU collected the data and had full access to the data and conducted the analyses. MU, ES, TF, RC, KP, and AH drafted the initial manuscript. All authors critically reviewed, provided feedback on the initial version of the manuscript, and approved the final version of the manuscript.
